# Impact of peptide permeation enhancer on tight junctions opening cellular mechanisms

**DOI:** 10.1016/j.bbrep.2022.101375

**Published:** 2022-10-27

**Authors:** Joël Brunner, Domitille Schvartz, Aurélie Gouiller, Alexandre Hainard, Gerrit Borchard

**Affiliations:** a School of Pharmaceutical Sciences, University of Geneva, Geneva, Switzerland; b Proteomics Core Facility, Faculty of Medicine, University of Geneva, Geneva, Switzerland

**Keywords:** Tight junction, PKC zeta, L-R5 peptide, Occludin, Protein interaction

## Abstract

The myristoylated pentapeptide, L-R5, contains an amino acid sequence of the zeta inhibitory peptide (ZIP) portion (pseudosubstrate) of protein kinase C zeta (PKC ζ). As PKC ζ is involved in the modulation of epithelial tight junctions (TJs) through the phosphorylation of TJ proteins, L-R5 was suggested to interact with the enzyme resulting in the enhancement of paracellular permeability. This study shows that L-R5 does not bind to the enzyme but interacts directly with TJ proteins. We show here that the binding of PKC ζ to occludin and its successive phosphorylation is prevented by L-R5, which leads to TJ disruption and enhanced epithelial permeability. Although L-R5 did not show any *in vitro* cytotoxicity, a proteomics study revealed that L-R5 interferes with other regulatory pathways, e.g., apoptosis and immune response. We suggest that structural modification of the peptide may increase the specificity TJ protein-peptide interaction.

## Highlights

• Microscale thermophoresis (MST) showed robust results for protein bindings.
• The competitivity of L-R5 peptide for the binding of occludin-PKC zeta was shown.
• Tight junctions proteins expression was decreased due to L-R5 peptide.
• Multiple other mechanisms can be explored to use L-R5 for other therapies.

## 1 Introduction

**Fig. 1:**
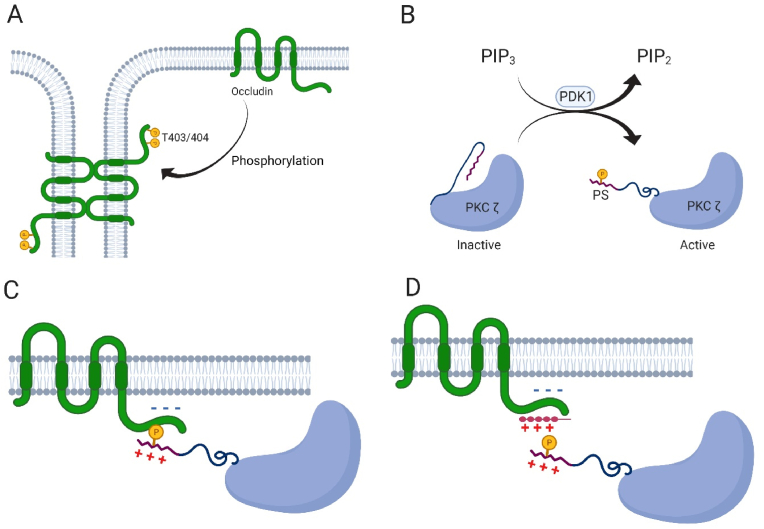
A: Pathway of Tight junction protein occludin activation by phosphorylation of 2 threonines. B: PP2A pathway of protein kinase C ζ (PKC ζ) activation by removing pseudosubstrate (PS) from the phosphorylation pocket. C: Interaction between PKC ζ PS and occludin for activation of occludin through phosphorylation. D: Mechanism of inhibition of occludin phosphorylation by zeta inhibitory peptide (ZIP).

Tight junctions (TJs) are responsible for the closure of intercellular junctions, thereby modulating the paracellular permeability of epithelial cell layers. TJs are regulated by the interactions of numerous intercellular proteins, with many mechanisms being potentiated by external factors [1]. TJ proteins mainly include the claudins family [2], occludin [3,4] and *zonula occludens* (ZO) group of proteins [5], the expression and activation of which is regulated by many different pathways. It has been shown that phosphorylation of threonine and tyrosine residues of TJ proteins is required to close TJs (Fig. 1A) [6]. These different phosphorylation reactions do occur by protein kinase C (PKC) [7].

PKCs are a family of serine/threonine kinases [8] that are among the major regulatory enzymes [9] being responsible for the phosphorylation of these residues under certain stimuli [10,11]. PKCs are classified into three different subtypes, called conventional, novel and atypical PKCs [12], based on their respective activation pathway [13]. Atypical PKCs are activated by phosphatidylserine in the PP2A pathway (Fig. 1B) [14,15], whereas novel PKCs also require diacylglycerol, and the conventional subtype the presence of Ca^++^. It has been previously shown that an altered expression of PP2A resulted in a decreased expression of occludin [16]. PKCs are also activated by an internal conformational change. A part of the C1 domain, called pseudosubstrate (PS), has been shown to be involved in PKC activation [17]. In addition to the modulation of epithelial permeability, PKCs have been identified to be involved in many different cellular transduction pathways, such as apoptosis, secretion, or cellular proliferation [18]. Clinical trials have been conducted to inhibit PKCs α and β to prevent cancer development [19]. However, none of these inhibitors has been used in a commercial product due to a lack of improved clinical outcome caused by the application of such inhibitors.

PKC ζ has been classified as an atypical PKC [20], which is mainly involved in cell proliferation and survival [12]. Its involvement in cell proliferation has been demonstrated in tumorigenesis [21]. Higher expression of this enzyme has been described in different cancer types and at different stages of tumour development. Examination of colorectal [22], lymphoid and respiratory tumours [23,24] revealed a connection between PKC ζ and tumour development. More specifically, PKC ζ appears to be involved in mitogenic signal transduction [25]. On the other hand, due to its involvement in cell proliferation, PKC ζ has also been shown to be overexpressed in advanced diabetes characterized by pancreatic hyperplasia [26] and to mediate insulin action by phosphorylation of the insulin receptor in adipose tissue [27].

The disruption of PKC ζ activity may trigger serious long-term problems. Activation of NFκB by this kinase is its main role in cell survival [21] and was shown to decrease cell death [19]. Inhibition of PKC ζ is also effective in reducing COPD symptoms [28] and in decreasing epithelial permeability [29]. This TJ modulation involves the enzyme via the Toll-like receptor 2 activation pathway. In addition, PKC ζ reduces epithelial permeability by phosphorylating TJ proteins occludin (threonines 403 and 404) and ZO-1 (serine residues) [29,30]. Inhibition of PKC ζ thus is considered to cause TJ opening and increase in epithelial permeability.

PKCs all contain a pseudosubstrate (PS) part regulating their activity. PS has been shown to keep the enzyme in an inactive state [32] by blocking its catalytic domain [33]. Activation of secondary messengers such as PDK1 releases inhibition of the PS and leaves the enzyme in an active form [34]. Exogenous and artificial peptides of an amino acid sequence resembling (parts of) the PS have been applied as PKC inhibitors [34]. The PKC ζ PS has been sequenced and is located between amino acids 113 and 129 [17]. A myristoylated peptide of a respective amino acid sequence named zeta inhibitory peptide (ZIP) is commercially available. It was initially thought that ZIP would directly inhibit PKC ζ by acting in the same way as PS and keeping the kinase in an inactive state. It has been then noted that ZIP was not specific and also decreased the activity of other PKCs [35]. Furthermore, ZIP still had an inhibitory effect in the absence of PKC ζ [36]. It has therefore been assumed that ZIP acted through a pathway other than through inhibition of PKC ζ [37]. A recent study showed that PS interacts electrostatically with the targeted proteins to form enzyme-protein complexes [38] coupled to PB1-PB1 (Phox and Bem1) interaction. The addition of ZIP to cellular processes does not directly inhibit PKC ζ but prevents the formation of this complex by competing with the PS of the enzyme (Fig. 1C + D). Phosphorylation cannot take place, nor can activation of occludin for example. Thus, ZIP does not inhibit the catalytic domain of PKC ζ [39].

In this study, the mechanism of TJ modulation in Caco-2 cells by inhibition of occludin phosphorylation was explored. A reduced myristoylated pentapeptide of ZIP, named L-R5, and several variations were tested previously and were shown to increase drug permeability through epithelial cell layers [40,41]. In this study we examined the mechanism by which L-R5 and its variations, as well as ZIP are able to increase epithelial cell permeability. Moreover, the influence of L-R5 on the expression of TJ proteins and their extent of phosphorylation were elucidated. Finally, a proteomic analysis on the implication of the peptide on other pathways was performed.

## 2 Materials and methods

### 2.1 Peptides and PKC ζ inhibitor

The different peptides L-R5 (myr-ARRWR [41]), D-R5 (amino acids in D form), myristoylated ZIP (13aa), L-R5 of a scrambled sequence (myr-WRARR) referred to here as Sc, and non-myristoylated L-R5 (referred to here as Wo) used in this study were obtained from Bachem AG (Bubendorf, Switzerland). The chemical PKC ζ inhibitor 5-(3-(*tert*-Butyl)-1-(3-chlorophenyl)-4,5-dihydro-1H-pyrazol-5-yl)-2-fluorophenol [42] (referred to here as In) was a gift by Dr. Engel (University of the Saarland, Germany).

### 2.2 Microscale thermophoresis (MST)

#### 2.2.1 Protein labelling

The protein construct of PKC ζ active (recombinant enzyme expressed in Sf21 insect cells) was purchased from Eurofins pharma (Dundee, United Kingdom) and the recombinant human C-terminal fragment of PKC ζ was purchased from RayBiotech (Peachtree Corners, GA, USA). Fluorescent labelling of both, PKC ζ and occludin was performed following the protocol of coupling the His-tag labelling kit RED-Tris-NTA 2nd generation (Nanotemper Technologies, Munich, Germany) to their respective histidine tail. The fluorescence of the tagged proteins was then measured with the monolith NT.115 microscale thermophoresis instrument (Nanotemper Technologies, Munich, Germany). An excitation LED of 100% was set for the Cap. Scan. The predicted optimal dilution was then calculated to obtain a final fluorescence signal of 100 Raw fluorescence [counts].

#### 2.2.2 Measurement of protein-peptide interactions

Hydrophilic capillaries were used for the different tests because the samples interacted with standard capillaries. The interaction between PKC ζ and occludin was established with serial dilutions of occludin in the assay buffer (Tris buffer pH 7.4) at concentrations between 0 and 35000 nM and a fixed concentration of 30 nM for PKC ζ. The interaction between PKC ζ and L-R5 was established with serial dilutions of the peptide in assay buffer at concentrations between 0 and 4000 μM and a fixed concentration of 20 nM for PKC ζ. The interaction between occludin and L-R5 was established with serial dilutions of L-R5 in the assay buffer at concentrations between 0 and 25000 μM and a fixed concentration of 50 nM for occludin. The interaction between PKC ζ and occludin in the presence of L-R5 at a sufficient concentration to bind to occludin was established with serial dilutions of occludin in assay buffer at concentrations between 0 and 11300 nM and a fixed concentration of 1.25 mM for L-R5 and 30 nM for PKC ζ. The final volume was 20 μl per dilution. For each experiment, a solution containing only the assay buffer was prepared as a negative control. The different solutions were analysed by MST at 20%, 40% and 80% MST power with a 100% LED intensity. The laser was switched on for 30 s and then switched off for 5 s. A repeat measurement was performed after 3 h of incubation in the capillaries and no significant changes in fluorescence intensity, *K*_d_ value and protein adsorption was noted. The results and *K*_d_ values were analysed by MO.affinity analysis 3 software (Nanotemper Technologies, Munich, Germany).

### 2.3 Cell culture

Caco-2 cells (ATCC, Manassas, VA, USA) were cultured in T75 flasks (Merck, Schaffhausen, Switzerland). The cells were incubated at 37 °C and a humidified atmosphere containing 5% of CO_2_. The medium containing 10% fetal bovine serum (FBS, Thermofisher, Waltham, MA, USA) was changed every 2–3 days. The Caco-2 cells were passaged every 5 days. The passage numbers were between 33 and 36.

For Western blot experiments, cells were seeded in 12-well plates (Merck, Schaffhausen, Switzerland) at a density of 50′000 cells/cm^2^. For the proteomic experiments, the cells were seeded in T10 flasks (Merck, Schaffhausen, Switzerland) at the same initial seeding density. After 7 days and two washes with warm phosphate buffered saline (PBS, Thermofisher, Waltham, MA, USA), the conditions were applied for 1 h. Peptides were applied at a concentration between 20 and 100 μM. A chemical inhibitor of PKC ζ was also tested. The molecule 4f [42] was used at a concentration of 10 μM in 0.1% DMSO as a positive control. All dilutions were done in 0.9% saline solution. After incubation, cells for Western blot experiments were lysed with RIPA buffer (Cell signalling, Danvers, MA, USA) containing protease and phosphatase inhibitors (Roche, Basel, Switzerland). After centrifugation to discard cell components, the samples were stored at −20 °C. Cells for proteomic analysis were detached with trypsin, washed 3 times with PBS and kept at −80 °C until analysis. Samples for proteomic analysis were produced in triplicate for each condition.

### 2.4 Immunoprecipitation

Specific immunoprecipitation of threonine phosphorylated proteins was performed using the immunoprecipitation kit from Abcam (ab206996, Cambridge, United Kingdom). The lysates used were the same as those described above. The protocol was scrupulously followed with the adaptations described by Wang et al. [43]. For 400 μg of proteins, 30 μg of specific threonine phosphorylated antibody (13–9200, Thermofisher, Waltham, MA, USA) was mixed with 30 μl of A/G sepharose beads. Separation of the proteins, antibody and beads was performed by adding the SDS loading buffer (Laemmli buffer, Bio-Rad, Hercules, CA, USA). The immunoprecipitated proteins were used directly in electrophoresis gel.

### 2.5 Immunoblot analysis

50 μg cell extracts and p-threonine proteins were separated by SDS-polyacrylamide gel electrophoresis (4–15%) (Bio-Rad, Hercules, CA, USA) and transferred to nitrocellulose membrane. Proteins of interest on the membrane were bound to primary antibodies (anti-occludin, anti-ZO-1, anti-F-actin, anti-PKC ζ, Cell signalling technology, Danvers, MA, USA) overnight at 4 °C with gentle agitation. These primary antibodies were then recognized by anti-mouse and anti-rabbit antibodies (Li-Cor Biosciences, Lincoln, NE, USA) and detected by Odyssey® imaging system (Li-Cor Biosciences). The signal of each protein was normalised to the signal of the corresponding actin.

### 2.6 Proteomic

#### 2.6.1 Sample preparation

Cell pellets were resuspended in 100 μL of 0.1% RapiGest Surfactant (Waters, Milford, MA, USA) in 50 mM ammonium bicarbonate (AB). Samples were heated for 5 min at 100 °C. Lysis was performed by sonication (6 × 30 s) at 70% amplitude and 0.5 pulse. Samples were kept 30 s on ice between each cycle of sonication. Samples were centrifuged for 10 min at 14′000×*g*. Protein concentration was measured by Bradford assay and 25 μg of each sample was subjected to protein digestion as follows: the sample volume was adjusted to 100 μL with 0.1% RapiGest in 50 mM AB. 2 μL of 50 mM dithioerythritol (DTE) were added and the reduction was carried out at 60 °C for 1h. Alkylation was performed by adding 2 μL of iodoacetamide 400 mM during 1 h at room temperature in the dark. Overnight digestion was performed at 37 °C with 5 μL of freshly prepared trypsin (Promega AG, Dübendorf, Switzerland) in 50 mM AB at a concentration of 0.2 μg/μL. To remove RapiGest, samples were acidified with trifluoroacetic acid (TFA), heated at 37 °C for 45 min and centrifuged 10 min at 17′000×*g*. Supernatants were then desalted with a C18 microspin column (Harvard Apparatus, Holliston, MA, USA) according to the manufacturer's instructions, completely dried under speed-vacuum and stored at −20 °C.

#### 2.6.2 ESI-LC-MS/MS

Samples were diluted at 1 μg/μL with loading buffer (5% CH_3_CN, 0.1% FA). Biognosys iRT peptides were added to each sample and 2 μg of peptides were injected onto the column. LC-ESI-MS/MS was performed on an Orbitrap Fusion Lumos Tribrid mass spectrometer (Thermo Scientific, San Jose, CA, USA) equipped with an Easy nLC1200 liquid chromatography system (Thermo Scientific, San Jose, CA, USA). Peptides were trapped on an Acclaim pepmap100, C18, 3 μm, 75 μm × 20 mm nano trap-column (Thermofisher) and separated on a 75 μm × 500 mm, C18 ReproSil-Pur (Dr. Maisch GmbH, Ammerbuch, Germany), 1.9 μm, 100 Å, custom-made column. The analytical separation was run for 135 min using a gradient of H_2_O/FA 99.9%/0.1% (solvent A) and CH_3_CN/H_2_O/FA 80.0%/19.9%/0.1% (solvent B). The gradient was run from 8% B to 28% B in 110 min, then to 42% B in 25 min, then to 95%B in 5 min with a final stay of 20 min at 95% B. Flow rate was of 250 nL/min a total run time was of 160 min. Data-Independent Acquisition (DIA) was performed with MS1 full scan at a resolution of 60,000 (FWHM) followed by 30 DIA MS2 scan with fix windows. MS1 was performed in the Orbitrap with an automatic gain control (AGC) target of 1 × 10^6^, a maximum injection time of 50 ms and a scan range from 400 to 1240 *m/z*. DIA MS2 was performed in the Orbitrap using higher-energy collisional dissociation (HCD) at 30%. Isolation window was set to 28 *m/z* with an AGC target of 1 × 10^6^ and a maximum injection time of 54 ms.

#### 2.6.3 Data analysis

DIA raw files were loaded into Spectronaut v.15 (Biognosys, Schlieren, Switzerland) and analysed by directDIA using default settings. Briefly, data were searched against the human Reference Proteome database (Uniprot, 2018–06, 21044 entries). Trypsin was selected as the enzyme, with one potential missed cleavage. Variable amino acid modification was oxidized methionine. Fixed amino acid modification was carbamidomethyl cysteine. Both peptide precursor and protein FDR were controlled at 1% (*Q value* < 0.01). Single Hit Proteins were excluded. For quantitation, Top 3 precursor area per peptides were used, “only protein group specific” was selected as proteotypicity filter and normalization was set to “global normalization”. The quantitative analysis was performed with MapDIA tool, using the precursor quantities extracted from Spectronaut output. No further normalization was applied. The following parameters were used: min peptides = 2, max peptides = 10, min correl = −1, Min_DE = 0.01, max_DE = 0.99, and experimental_design = replicate design. Proteins were considered to have significantly changed in abundance with an FDR ≤0.05 and an absolute fold change FC≥ |1.5| (log2FC ≥ |0.58|).

## 3 Results

### 3.1 L-R5 decreases the affinity between PKC ζ and occludin

Microscale thermophoresis is a method used to quantify the affinity between two molecules (e.g., proteins) by increasing the concentration of the ligand while keeping the concentration of the target fixed. The fluorescence during the analysis time will be higher overall and by plotting all results, the dissociation constant *K*_d_ can be calculated. This constant was calculated using equation (1) [44]:

(1) $$K_{d}=\frac{{\left[A\right]}^{x}\times {\left[B\right]}^{y}}{\left[A_{x}B_{y}\right]}\;when\;A_{x}B_{y}\underset{\Leftrightarrow }{\;}\;xA+yB$$

with A and B, the 2 components (ligand, target) interacting and x and y the 2 stoichiometric factors.

**Fig. 2:**
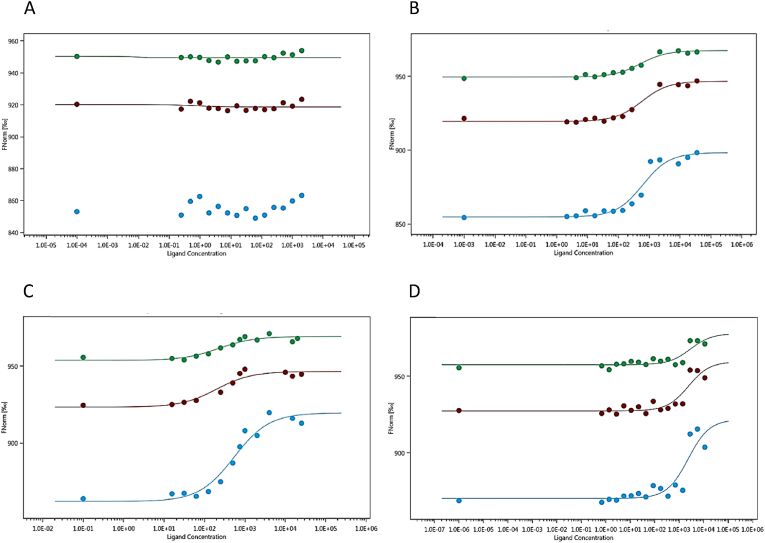
Microscale thermophoresis (MST) measurements, green, red and blue lines represent the same samples measured at respectively 20%, 40% and 80% MST intensity. A: interaction of labelled protein kinase C ζ (PKC ζ) (20 nM) and L-R5 as ligand (0–4 mM); B: interaction of labelled PKC ζ (30 nM) and occludin as ligand (0–35 μM); C: interaction of labelled occludin (50 nM) and L-R5 as ligand (0–25 mM); D: interaction of labelled PKC ζ (30 nM), L-R5 as competitor (1,25 mM) and occludin as ligand (0–11,3 μM). n = 2 but only n = 1 is shown due to restrictions of the software. (For interpretation of the references to color in this figure legend, the reader is referred to the Web version of this article.)

First, the different binary interactions were tested between PKC ζ, occludin and L-R5. The myristoylated peptide L-R5 showed no interaction with the enzyme (Fig. 2A), as no difference of fluorescence over time was noted in the heated region for this solution, even at a concentration of 4 mM for L-R5. On the other hand, occludin interacts with both PKC ζ and L-R5 (Fig. 2B and C, respectively). An increase in normalised fluorescence over time reveals the formation of a protein-enzyme and protein-peptide complex. The *K*_d_ measured for these interactions are 617,33 ± 97,4 nM and 605,67 ± 34,2 μM, respectively. In Fig. 2D, the interaction between PKC ζ and occludin still occurs but at a higher *K*_d_ (2,16 ± 0,59 μM), possibly due to the presence of L-R5.

### 3.2 TJ proteins expression is decreased by L-R5 and analogues

**Fig. 3:**
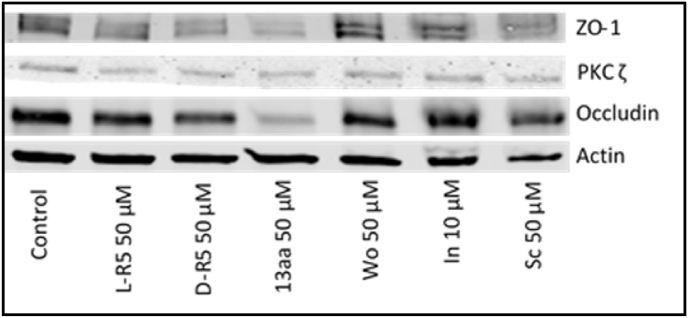
Total protein extracts from Caco-2 cells were immunoblotted for zonula occludens (ZO-1), protein kinase C ζ (PKC ζ), occludin and actin after different conditions applied for 1 h: Control (Medium), L-R5 50 μM, D-R5 50 μM, 13aa 50 μM, without myristoyl (Wo) 50 μM, inhibitor (In) 10 μM and scrambled (Sc) 50 μM.

Different peptides and a chemical inhibitor of PKC ζ were applied on Caco-2 cells. The expression of TJ proteins was then quantified by immunoblotting. First, the expression of PKC ζ was not affected by any condition (Fig. 3). On the other hand, expression of both occludin and ZO-1 were decreased by the myristoylated peptides. The unmyristoylated peptide Wo and the chemical inhibitor In showed no effect on their expression.

**Fig. 4:**
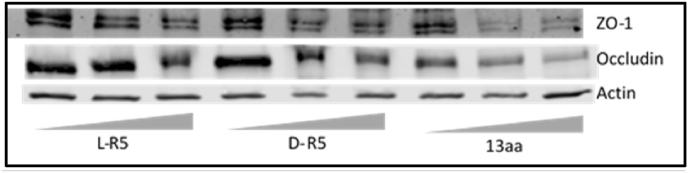
Total protein extracts from Caco-2 cells were immunoblotted for ZO-1, occludin and actin after different gradient conditions for 1 h: L-R5, D-R5 and 13aa at 20, 50 and 100 μM.

**Fig. 5:**
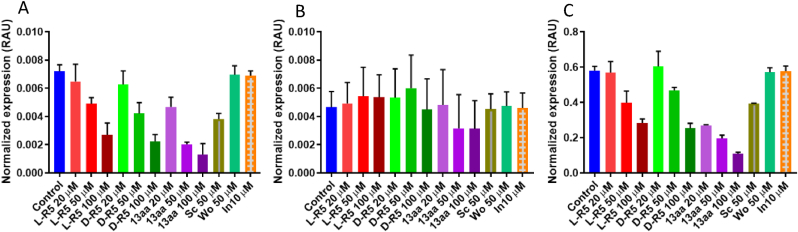
Graphical representation of Western blots results for A: zonula occludens (ZO-1), B: protein kinase C ζ (PKC ζ), and C: occludin after different conditions applied for 1 h: Control (Medium), L-R5 50 μM, D-R5 50 μM, 13aa 50 μM, without myristoyl (Wo) 50 μM, inhibitor (In) 10 μM and scrambled (Sc) 50 μM normalised with actin's expression. n = 3.

To confirm these results, solutions of L-R5, D-R5 and 13aa peptides were applied on the cells at different concentrations to demonstrate the concentration dependence of TJ protein expression (Fig. 4). As the concentration of the peptides increased, the expression of ZO-1 and occludin decreased. In Fig. 4, the peptide 13aa is shown to impart a greater reduction of TJ protein expression. The enzyme PKC ζ was not blotted as the peptides were shown to have no effect on its expression. Fig. 5 confirms these results with a normalised quantification of TJs proteins by the expression of actin.

### 3.3 Active occludin expression is not affected by ZIP derivatives

**Fig. 6:**
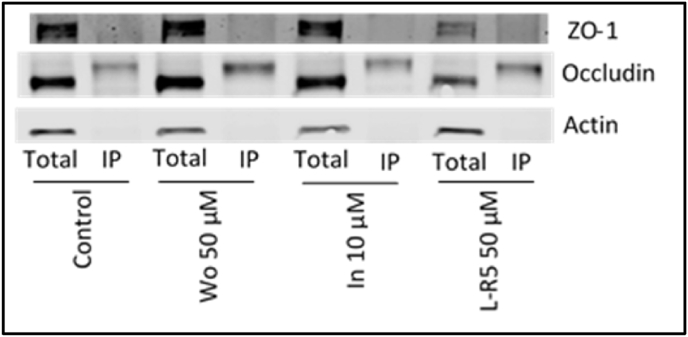
Total protein extracts and immunoprecipitated phospho-threonine protein extracts (IP) were immunoblotted for ZO-1, occludin and actin under different conditions after 1 h: Control (medium), Wo 50 μM, In 10 μM and L-R5 50 μM.

**Fig. 7:**
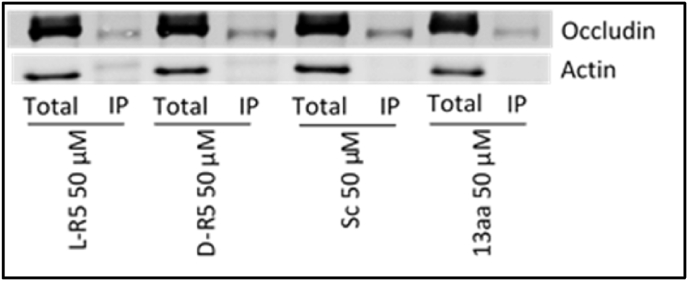
Total protein extracts and immunoprecipitated phospho-threonine protein extracts (IP) were immunoblotted for occludin and actin under different conditions after 1 h: L-R5 50 μM, D-R5 50 μM, Sc 50 μM and 13aa 50 μM.

**Fig. 8:**
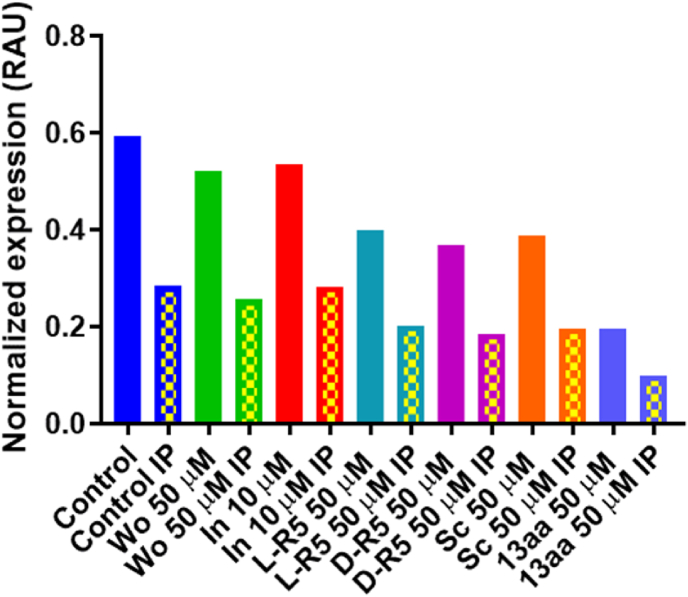
Graphical representation of Immunoprecipitated proteins with threonine specific antibody Western blots results for occludin after different conditions applied for 1 h: Control (Medium), L-R5 50 μM, D-R5 50 μM, 13aa 50 μM, without myristoyl (Wo) 50 μM, inhibitor (In) 10 μM and scrambled (Sc) 50 μM normalised with actin's expression. n = 1.

It was confirmed that the total amount of ZO-1 and occludin is reduced by the application of ZIP designed peptides (Fig. 3, Fig. 4). As ZIP is supposed to block the phosphorylation of occludin by PKC ζ, the total amounts of TJ proteins and threonine-phosphorylated TJ proteins were compared (Fig. 6, Fig. 7). The absence of ZO-1 as well as that of actin can be noted in the immunoprecipitated samples (Fig. 5). The occludin signal is still only decreased in the presence of L-R5, but not in the presence of both Wo and In. For immunoprecipitated phospho-threonine occludin, the signal between the different conditions is conserved, no significant difference in the expression of p-occludin was found. The same results are shown in Fig. 6 where the amount of total occludin as well as p-occludin is the same under all conditions. The graphical results (Fig. 8) shows that independently to the condition applied, the active occludin (immunoprecipitated) is kept at a ratio of the half of total occludin.

### 3.4 The scope of L-R5 is wider than expected

**Fig. 9:**
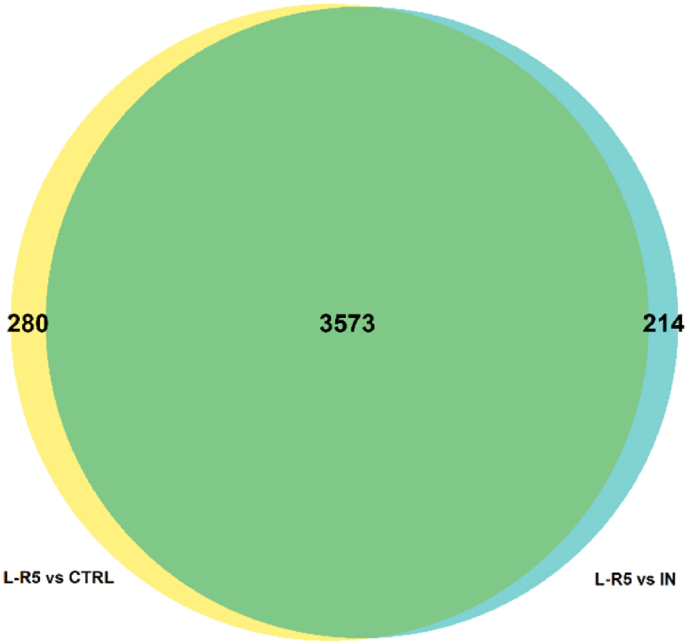
Venn diagram of the significantly regulated proteins in each comparison L-R5 vs control (CTRL) and L-R5 vs inhibitor (In). In total, 4067 proteins were significantly regulated with high confidence (LFDR≤0.05). n = 3.

**Fig. 10:**
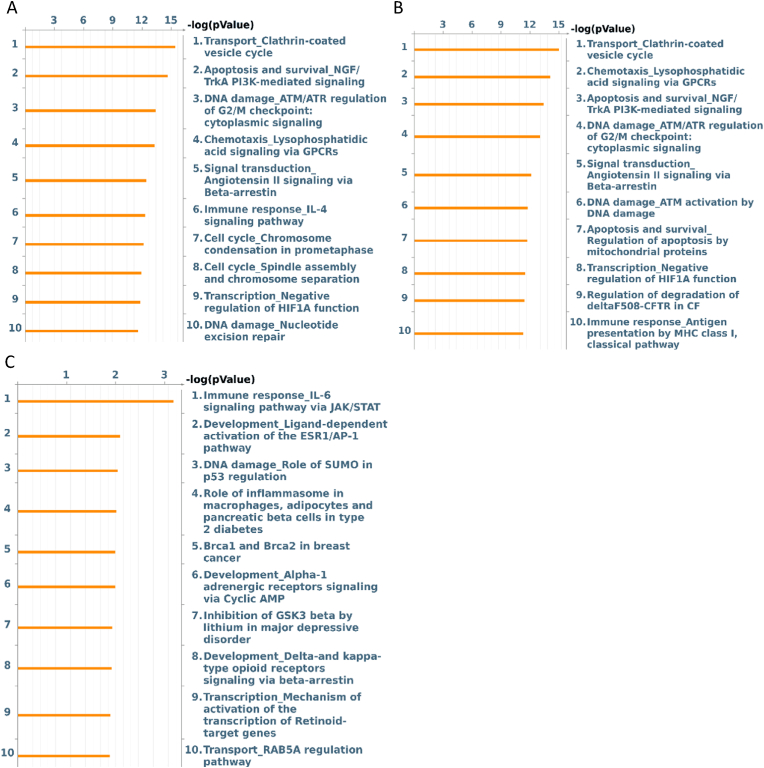
Proteomic pathway analysis (Top 10) as determined by MetaCore analysis. Pathways are listed in order of statistical significance. Orange bars represent the -log(*p*-value) of proteomics analysis. A: Top 10 pathways affected by control versus L-R5 comparison. B: Top 10 pathways affected by inhibitor (In) versus L-R5 comparison. C: Top 10 pathways affected by control versus In comparison. n = 3. (For interpretation of the references to color in this figure legend, the reader is referred to the Web version of this article.)

Quantitative proteomic analysis of the different conditions allowed for the identification and quantification of 5115 proteins. By combining data of significantly differently expressed proteins in the control group versus L-R5 treated group (3787 proteins) and In versus L-R5 (3853 proteins), a total of 3573 proteins were found in common between these two comparisons (Fig. 9). 280 proteins were specifically differentially expressed only in the control versus L-R5 comparison, and 214 only in the In versus L-R5 comparison. Top 10 pathways influenced in each binary comparison were reported using MetaCore software analysis (Fig. 10). 6 pathways of 10 were common in control versus L-R5 and In versus L-R5, but none with In versus control. The significance level of affected pathways is represented by their respective -log(*p*-value).

**Fig. 11:**
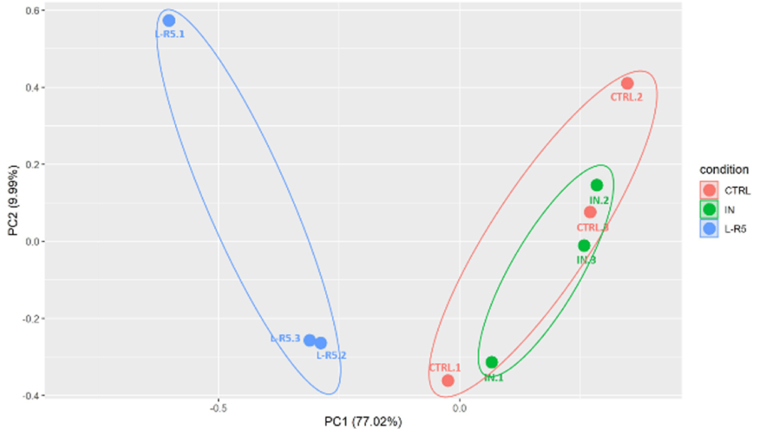
PCA clustering analysis plot of proteins from Caco-2 cells mediated by 3 conditions: control (red), L-R5 50 μM (blue) and inhibitor (In) 10 μM (green). The protein expression profiles were visualized by using the extended data analysis module described in 2.6.3. n = 3. (For interpretation of the references to color in this figure legend, the reader is referred to the Web version of this article.)

Global relationships among the different conditions were represented by a principal component analysis (PCA) as depicted in Fig. 11. PCA based on protein level of each replica (represented by a spot), underlines that 77% of the dataset variability is carried by the first principal component and separates the L-R5 group from the two other ones. The second source of variability (PC2) is lower (9.99%) and does not tend to separate sample groups.

PCA demonstrated that the control and In conditions grouped the same proteins but L-R5 grouped completely different expressed proteins. For example, the expression of the protein IRS1 (insulin receptor substrate) was decreased in the comparison L-R5 versus control and L-R5 versus In, but not in the comparison In versus control. In another example, the expression of proteins RICH1 (Rho GTPase-activating protein 17) and CDC 42 (cell division control protein 42 homolog) was increased by L-R5, but not by In alone.

**Fig. 12:**
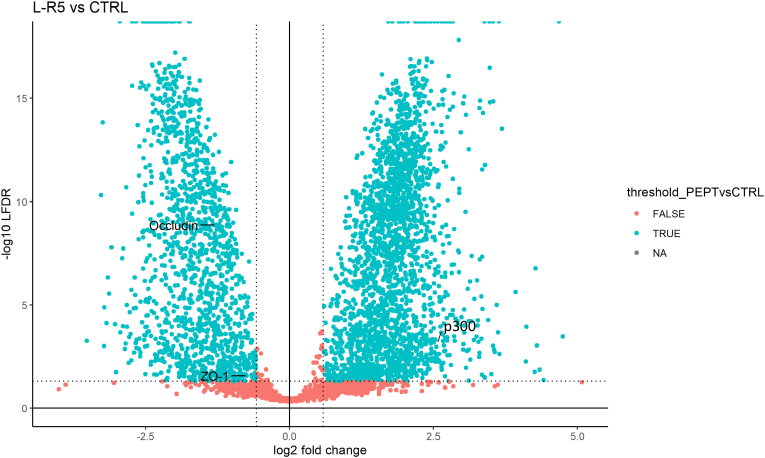
Volcano plot representing log2 fold-change and –log10 LFDR of all the proteins from Caco-2 cells quantified in the comparison L-R5 50 μM vs control (CTRL). Blue dots represent significantly different proteins, which are above LFDR threshold ≤0.05) and fold-change threshold (log2FC ≥ |0.58|). Proteins p300, occludin and zonula occludens (ZO-1) have been highlighted. n = 3. (For interpretation of the references to color in this figure legend, the reader is referred to the Web version of this article.)

**Fig. 13:**
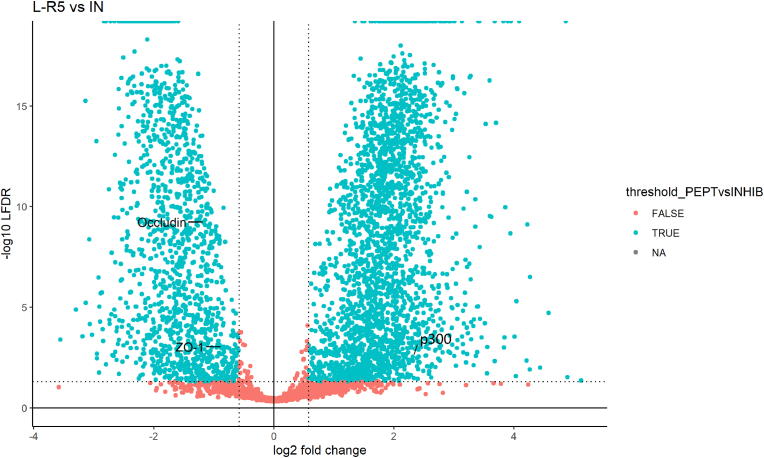
Volcano plot representing log2 fold-change and –log10 LFDR of all the proteins from Caco-2 cells quantified in the comparison L-R5 50 μM vs inhibitor (In) 10 μM. Blue dots represent significantly different proteins, which are above LFDR threshold ≤0.05) and fold-change threshold (log2FC ≥ |0.58|). Proteins p300, occludin and zonula occludens (ZO-1) have been highlighted. n = 3. (For interpretation of the references to color in this figure legend, the reader is referred to the Web version of this article.)

**Fig. 14:**
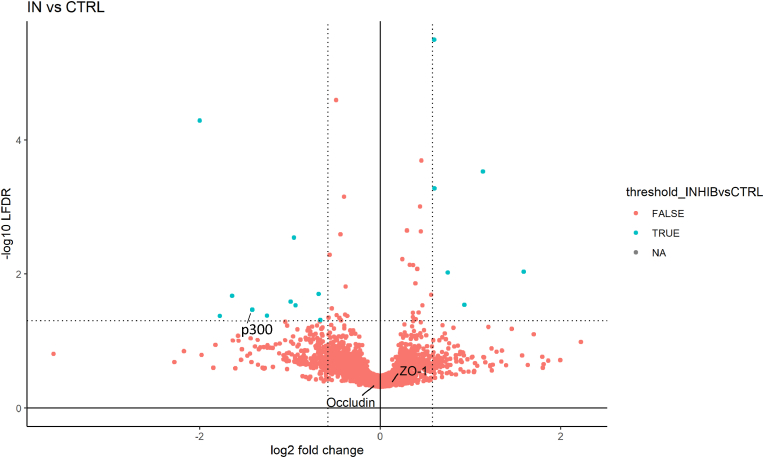
Volcano plot representing log2 fold-change and –log10 LFDR of all the proteins from Caco-2 cells quantified in the comparison inhibitor (In) 10 μM vs control (CTRL). Blue dots represent significantly different proteins, which are above LFDR threshold ≤0.05) and fold-change threshold (log2FC ≥ |0.58|). Proteins p300, occludin and zonula occludens (ZO-1) have been highlighted. n = 3. (For interpretation of the references to color in this figure legend, the reader is referred to the Web version of this article.)

To highlight differentially expressed proteins, a binary comparison between all conditions of downregulated and upregulated proteins was presented in the form of volcano plots (Fig. 12, Fig. 13, Fig. 14). The assessment of differential expression of protein levels was done by ANOVA test (cut-off at *p*-value *p* ≤ 0.05). 3787 proteins were considered to be significantly differentially expressed between control and L-R5 conditions. Among these, 2384 were upregulated (log 2 (FC) ≥ 0) and 1403 downregulated (log 2 (FC) < 0). 3853 proteins were considered differentially expressed when comparing In and L-R5 conditions. Among these, 2488 were upregulated (log 2 (FC) ≥ 0) and 1365 downregulated (log 2 (FC) < 0). 16 proteins were considered differentially expressed when comparing control and In conditions. Among these, 6 were upregulated (log 2 (FC) ≥ 0), and 10 downregulated (log 2 (FC) < 0).

## 4 Discussion

Initially, synthetic PSs (ZIP for PKC ζ) were used as inhibitors of their respective PKC isoforms [34]. However, given the results presented above, the inhibition of PKC ζ does not occur by interaction with the enzyme directly but with the target proteins of this kinase (Fig. 2A–C). Even if the experimental environment is not the same as in the cytoplasm, the comparison of different strength of interactions between ligands can be done in the same conditions. MST analysis provided *K*_d_ values, however, it would also be useful to understand whether the interaction between L-R5 and occludin is reversible by determining K_on_ and K_off_ of this interaction [45]. Furthermore, upon binding to occludin, results showed that L-R5 decreased the affinity between PKC ζ and the TJ protein (Fig. 2D), with *K*_d_ values being significantly increased. ZIP is therefore not a direct inhibitor of PKC ζ but acts through competitive binding to the target protein, inhibiting its phosphorylation. The peptide structures may therefore be called "zeta competitor peptides" (ZCPs). Preliminary studies have shown that this interaction is electrostatic in nature due to the positive charges of the arginines of the ZCP [38]. This is confirmed by the same effects seen with the administration of L-R5, D-R5 and Sc (Fig. 3), with the electrostatic interaction being identical for these three peptides given their equal overall charge. 13aa more strongly decreases TJ protein expression. Its positive charge and its higher complementarity to the target protein provides greater affinity and probably stronger competitive inhibition. However, the peptide without its myristoylated hydrophobic tail can no longer enter the cell [41,46]. Inhibition of PKC ζ by In did not result in a significant decrease in TJ protein expression. Obviously, the specific inhibition is not sufficient to have an impact on TJ modulation and decrease expression of TJ proteins.

By competing with the enzyme for binding to occludin, L-R5 prevents phosphorylation of the protein and thus its activation. This prevents successively the closure of TJs [47,48]. ZIP has already been shown to decrease the expression of certain TJ proteins such as occludin and ZO-1 [30]. The ZIP-derived peptide L-R5 has also been shown here to have the same effect (Fig. 3, Fig. 4, Fig. 12, Fig. 13). Logic would suggest that the cell increases the production of blocked proteins to compensate for this inhibition. Otherwise, this decrease could be due to i) a tendency of the cell to maintain the same ratio of phosphorylated/non-phosphorylated proteins [49], ii) a recycling of blocked proteins, detected as defective [50,51], or iii) a degradation of these proteins because L-R5 modifies them and makes them unusable or uneffective.

ZO-1 could not be quantified (Fig. 6) because this protein is phosphorylated at a serine residue [31] and was therefore eliminated during immunoprecipitation. It has previously been shown that inhibition of occludin phosphorylation triggers a decrease in its expression [52]. The results presented above (Fig. 6, Fig. 7) do not allow the same conclusions to be drawn. Indeed, the expression levels of occludin did not change significantly under the conditions applied in this study. This may be due to an insufficient purification and quantification method, but it may also be likely that phosphorylated occludin (p-occludin) expression does not change. Through the activity of the peptide, the total intracellular concentration of occludin is reduced. In response, the biosynthesis of occludin is activated by the cell. Ideally, the effect of the peptides on all types of occludin should be quantified under each condition, including occludin, threonine-phosphorylated, tyrosine-phosphorylated, serine-phosphorylated and non-phosphorylated occludins. Moreover, It seems the cells keep a constant ration of active/total occludin (Fig. 8). Even in the case of a change in proteins expression the ratio is kept probably to avoid any disequilibrium. However, Rao speculated that activation of occludin by serine or threonine phosphorylation would not have the same function [53]. Further investigations are still needed to fully unravel the mechanics of occludin phosphorylation inhibition and its consequences.

Previous studies have shown that ZIP is not specifically and competitively inhibiting PKC ζ phosphorylation of some TJ proteins [37] (occludin and ZO-1). The structural similarities of the PS of the PKC family [24] does explain this non-specificity. Therefore, L-R5 would also be competitive with other PKCs and would interfere with most intracellular mechanisms involving PKCs. The PCA results presented here show that the expression of many proteins is altered by the presence of L-R5 (Fig. 11), in contrast to In, for which the results are similar to the control condition. Given the absence of cytotoxicity of L-R5 as shown in previous studies [41], this wide range of potential interaction of the peptide was not expected. It may not even translate into safety issues *in vivo*, however, greater specificity of the peptide for selected TJ proteins could avoid a potential risk.

In this broad field of action, the expression of occludin and ZO-1 proteins is significantly decreased by L-R5 (Fig. 12, Fig. 13), as previously demonstrated by Western blots. In contrast, In has no impact on their expression (Fig. 14). Inhibiting PKC ζ alone is not sufficient to decrease their expression, but possibly increase permeability. Concerning L-R5, the volcano plots highlighting the effects of L-R5 (Fig. 12, Fig. 13) are similar. The proteins affected by the peptide are essentially the same for control and for In (Fig. 9), and only a few proteins are affected by In condition compared to control. It was mentioned earlier that occludin and ZO-1 are affected by the peptide, but the results also show that claudins 1 and 4 are not. This may be explained by the fact that these proteins are not regulated by PKC ζ [30] and L-R5 is still specific to TJ proteins phosphorylated by PKC ζ, and not all TJ proteins. Furthermore, the expression of occludin and ZO-1 is significantly decreased (Fig. 12, Fig. 13), which would explain the opening of TJs by L-R5 in addition to the phosphorylation inhibition.

As a proof of concept for L-R5, the expression of PKC ζ-related proteins other than TJ proteins were analysed. In a mechanism of inflammation, the phosphorylation of JAK1 (Janus kinase 1) is mediated by PKC ζ [54]. JAK1 expression is not influenced by L-R5, but its activity is decreased, which is shown by the decreased expression of its target protein, IRS1 [55]. JAK1 phosphorylates this receptor. As for occludin, the activity of the PKC ζ target protein is inhibited by the pentapeptide.

In another intracellular mechanism, PKC ζ is responsible for the phosphorylation of PARD3 (Partitioning defective 3 homolog protein) [56]. This protein is then thought to bind to angiomotin [57]. This complex then inhibits the RICH1 protein [58]. The increase in RICH1 expression observed in our study is due to the inhibition of the activity of the angiomotin-PARD3 complex by L-R5. PARD3 is neither phosphorylated nor activated by PKC ζ. The influence of L-R5 on the angiomotin-PARD3 complex confirms the activity of L-R5 on other than those mediated by PKC ζ. In contrast, no similar results were found in the In versus control condition. The inhibition of PKC ζ by In appears to be too weak or non-existent.

Using the MetaCore software, the 10 most enriched pathways in the different comparisons are listed in Fig. 10. Pathways enriched in conditions where L-R5 was added (Fig. 10A and B) showed essentially the same results, whether compared to the control or under incubation with In. In contrast, the pathways significantly affected by the In versus control condition are all different from the first 2 conditions (Fig. 10C).

Only the pathways “chemotaxis lysophosphatidic acid signalling via GPCRs”, “apoptosis and survival NGF/TrkA PI3K-mediated signalling” and “immune response IL-4 signalling pathway” are pathways importantly influenced by L-R5 including PKC ζ. The L-R5 peptide therefore has a very strong influence on the disruption of intracellular mechanisms. This is confirmed by the important -log(*p*-values) of 12–15. The opening of TJs was not observed in these pathways despite the significant influence of L-R5 on this mechanism. It appears that not the regulation of TJs is the mechanism most affected by L-R5, but vesicle formation for intracellular transport (Fig. 10A). Another type of transport [59] is thus significantly stimulated by the presence of the peptide. In addition, the mechanism of PI3K-mediated apoptosis is also strongly affected by L-R5. As the involvement of PKC ζ in this pathway is proven [60], it is highly conceivable that the peptide interacts with the enzyme's contribution to this mechanism. One of the target proteins of the enzyme implicated in this pathway is GSK3β [61], whose expression is increased by the presence of the peptide. This results in a significant increase in cell survival [62]. Finally, the “chemotaxis lysophosphatidic acid signalling via GPCR” mechanism is also strongly affected by the peptide. GSK3β linked to PKC ζ is again involved in this pathway [63]. In addition, a decrease in cell proliferation and in formation of adherens junctions, which are complementary junctions to TJs, is observed [64]. Both mechanisms are involved in cancer progression. These results could explain the link between the inhibition of PKCs and the reduction of tumour progression [21].

In is considered as an inhibitor of PKC ζ, but none of the top 10 pathways influenced in the comparison In versus control include PKC ζ. The pathway “development delta- and kappa-type opioid receptors signalling via beta-arrestin” has been referenced as the only one in the list to include PKCs in its mechanism [65]. The effective inhibition of PKC ζ specifically by In is still to be proven *in vitro* or the doses have to be increased. In Fig. 10C, the common point of these pathways is the involvement of E1A binding protein p300, whose expression is significantly decreased by In. On the other hand, the expression of this protein is increased by the presence of L-R5. As this histone acetyltransferase is not linked to PKC ζ mechanisms [66], it would therefore appear that In interacts with other factors. Moreover, the pathways addressed by In do not imply PKC ζ. The interaction with the kinase is proven [42], but other targets have to be considered as well. However, in view of the wide range of interactions of the peptide, it is legitimate to ask whether the PS derivative modulates TJs by direct interaction, or whether this opening is a result of the sum of all mechanisms affected by the peptide.

The contradictory results between the influence of L-R5 and In can be explained by their different targets. In was synthesised to inhibit PKC ζ, whereas L-R5 blocks the activity of the enzyme by competition with the target proteins. The phosphorylation mechanism cannot be carried out by PKC ζ or even all PKCs. Therefore, the consequence of L-R5 is not visible on the expression of PKC ζ or their target proteins, but on the proteins secondarily linked with the enzyme. The lack of phosphorylation prevents the activity of the target protein. Occludin for example cannot be active in the presence of L-R5. The subsequent decrease in its expression is due to a negative feedback on the expression mechanism.

## 5 Conclusion

In this study, the interaction of L-R5, and by extension ZIP and PS of PKC ζ, and PKC ζ with occludin was proven. Furthermore, L-R5 was shown to compete with PKC ζ when binding to occludin, which prevents enzyme-protein interaction. This binding could explain the reason for the opening of TJs in the presence of L-R5. Furthermore, the peptide and its analogues decrease the expression of TJ proteins, in contrast to a specific PKC ζ inhibitor. This decrease may be due to an uneffective modification of the protein. As L-R5 prevents binding between PKC ζ and occludin, the ratio of p-occludin to total occludin should decrease. However, this is not the case. The intracellular regulation of the balance between active and inactive occludin is disturbed by the presence of the peptide and the amount of p-occludin remains the same. The decrease in TJ protein expression was confirmed by proteomics. However, this study showed that L-R5 affects several other mechanisms than just TJ modulation. The peptide modulates many more pathways than those implicating PKC ζ. Finally, competition with PKC ζ is desirable for TJ opening, but this interaction needs more specificity for the TJ proteins involved.

## Declaration of competing interest

The authors declare that they have no known competing financial interests or personal relationships that could have appeared to influence the work reported in this paper.

## Data Availability

Data will be made available on request.
